# Mistreatment during facility-based childbirth in Benin City, Nigeria: A convergent mixed-methods study of prevalence and drivers

**DOI:** 10.1186/s12884-026-09541-z

**Published:** 2026-08-10

**Authors:** Debra Eluobaju, Friday Okonofua, Marika Toscano, Chi Chiung Grace Chen, Gelila Goba, Ahizechukwu Eke

**Affiliations:** 1 Department of Obstetrics and Gynecology, University of Benin Teaching Hospital, Benin City, Nigeria; 2 Department of Gynecology and Obstetrics, Johns Hopkins Hospital, 1800 Orleans Street, Baltimore, MD 21287 USA; 3 Department of Gynecology and Obstetrics, University of Miami Health Systems, Miami, USA

**Keywords:** Mistreatment, Childbirth, Facility-based delivery, Nigeria, Skilled birth attendants, Maternal health, respectful maternity care

## Abstract

**Background:**

Mistreatment during facility-based childbirth undermines maternal health outcomes and trust in health systems, particularly in low- and middle-income countries. Nigeria bears one of the highest global burdens of maternal mortality, yet limited large-scale mixed-methods studies have examined how mistreatment is both experienced by women and shaped by health system conditions. This study assessed the prevalence, forms, and drivers of mistreatment during childbirth in Benin City, Nigeria, integrating women’s and skilled birth attendants’ (SBAs) perspectives.

**Methods:**

We conducted a convergent parallel mixed-methods study between July–August 2024 across three healthcare facilities (tertiary, secondary, and private) in Benin City. Postpartum women (≤ 12 months since delivery) completed a 45-item mistreatment survey (*n* = 656). Mistreatment was categorized across eight domains. Descriptive statistics and chi-square analyses were performed. In-depth interviews were conducted with postpartum women (*n* = 20), and four focus group discussions were held with SBAs (*n* = 26). Qualitative data were analyzed using constructivist grounded theory. Findings were integrated using a joint display matrix to assess convergence, divergence, and complementarity.

**Results:**

Overall, 89.5% of women reported experiencing at least one form of mistreatment, though only 2.6% labeled their experience as mistreatment. The most common domains were undignified care (70%) and loss of autonomy (48.2%), followed by physical abuse (37.5%) and abandonment (16.3%). Vaginal delivery, higher education, and antenatal care attendance were significantly associated with reported mistreatment (*p* < 0.05).

Qualitative analysis identified five themes: divergent expectations between women and SBAs; shared labeling of laboring women as “uncooperative”; inconsistent definitions of mistreatment; provider burnout; and structural constraints including understaffing, limited infrastructure, and financial barriers. Both groups used the term “uncooperative” to describe laboring behavior, though with different interpretations. Women emphasized disrespect, lack of consent, and loss of autonomy, while SBAs contextualized care within systemic constraints and workload pressures. Integration showed strong convergence between quantitative autonomy violations and qualitative accounts of restricted companionship, hierarchical communication, and limited shared decision-making.

**Comclusions:**

Mistreatment during childbirth in urban Nigeria is highly prevalent and sustained by intersecting interpersonal and structural health system factors. Efforts to improve maternal health outcomes must address workforce capacity, institutional norms, accountability mechanisms, and rights-based antenatal education to ensure that clinical safety and respectful maternity care are jointly prioritized.

**Supplementary Information:**

The online version contains supplementary material available at https://doi.org/10.1186/s12884-026-09541-z.

## Introduction

Maternal mortality (MM) remains a major global health challenge, disproportionally affecting women in low – and middle-income countries (LMICs) [1, 2]. Although global MM rates have declined over three decades, progress has been uneven, with sub-Saharan Africa accounting for the majority of preventable maternal deaths [3–6]. Nigeria contributes substantially to this burden, with an estimated maternal mortality ratio of 993 deaths per 100,000 live births and nearly one-third of global maternal deaths in 2020 [6, 7]. Most maternal deaths result from preventable causes, including hemorrhage and hypertensive disorders, that are effectively managed through timely access to skilled obstetric care [1, 3, 5].

Beyond access to care, the quality of care during childbirth is now recognized as a critical determinant of maternal outcomes. Respectful maternity care (RMC) is a core global strategy for improving quality and preventing harm during facility-based childbirth [8, 9]. The World Health Organization defines RMC as care that maintains dignity, privacy, confidentiality, informed choice, and freedom from harm and mistreatment [10]. Within this framework, mistreatment during childbirth is not only a clinical quality failure but also a violation of fundamental human rights [11–13].

Mistreatment during childbirth; including physical, verbal, or emotional abuse; neglect; stigma and discrimination; non-consented care; and loss of autonomy constitutes a violation of women’s rights and undermines the provision of safe, equitable care [9, 11, 14, 15]. It has been explicitly identified by WHO as a key barrier to achieving universal respectful maternity care and improving maternal health outcomes. Importantly, mistreatment contributes directly to delays in seeking and utilizing facility-based childbirth services, thereby increasing the risk of preventable maternal morbidity and mortality [9, 15]. Women who anticipate or experience disrespectful care are less likely to return to health facilities for subsequent deliveries, reinforcing cycles of poor outcomes [9, 11, 14, 15].

Global evidence demonstrates that mistreatment during childbirth is widespread. A systematic review by Bohren et al. documented multiple forms of mistreatment across diverse settings, while the WHO multi-country study reported that more than one-third of women experience at least one form of mistreatment during facility-based childbirth [16, 17]. However, prevalence estimates vary widely due to differences in definitions, measurement approaches, and sociocultural norms influencing reporting.

In sub-Saharan Africa and Nigeria specifically, studies consistently report high levels of verbal abuse, neglect, non-consented care, and poor communication during childbirth [18–21]. However, most available studies are facility-specific or regionally limited, and national-level estimates remain scarce. Reported prevalence varies widely across settings, highlighting the need for context-specific epidemiological evidence as well as deeper understanding of underlying drivers [19, 20, 22–25] .

Mistreatment during childbirth is driven by multilevel determinants. At the interpersonal level, provider behaviors may be shaped by attitudes, biases, and communication practices [26, 27]. At the institutional level, understaffing, overcrowding, lack of essential supplies, weak accountability mechanisms, and poor infrastructure create conditions where respectful care is difficult to maintain [28, 29]. At the structural level, gender inequality, hierarchical power dynamics, and sociopolitical inequities normalize poor treatment of women in health systems [30]. Accordingly, mistreatment is increasingly understood as a systems-level phenomenon rather than an individual behavioral issue.

Despite growing recognition of mistreatment as a global health priority, few studies in Nigeria have used mixed-methods designs integrating both women’s and skilled birth attendants’ perspectives. This limits understanding of how mistreatment is experienced, normalized, and sustained within health systems.

This study therefore examines the prevalence, forms, and drivers of mistreatment during facility-based childbirth in Benin City, Nigeria, using a convergent parallel mixed-methods design. By integrating quantitative epidemiological estimates with qualitative insights, the study aims to provide a comprehensive understanding of mistreatment as a health system phenomenon and to identify actionable strategies for improving respectful maternity care in similar LMIC settings.

## Methods

### Study design and setting

A convergent parallel mixed-methods design was used, with simultaneous collection and equal prioritization of quantitative and qualitative data. This approach allowed examination of mistreatment from both women’s and SBAs’ perspectives while strengthening validity through triangulation [31].

The study was conducted between July and August 2024 in Benin City, the capital of Edo State in southern Nigeria, with an estimated metropolitan population exceeding 1.5 million. Nigeria’s health system is organized into primary, secondary, and tertiary levels of care [32]. As a major urban referral center, Benin City contains a high concentration of secondary- and tertiary-level facilities serving women from Edo State and neighboring regions [33, 34].

Three facilities were purposively selected to capture variation in ownership and level of care: University of Benin Teaching Hospital (UBTH), a federal tertiary referral hospital; Edo Specialist Hospital, a state-owned secondary hospital; and St. Philomena Catholic Hospital, a private faith-based hospital. Together, these facilities represent the principal public tertiary, public secondary, and private obstetric care settings in Benin City.

Women were recruited through child immunization clinics within the participating hospitals. Immunization clinics were selected because they represent a routine point of postpartum contact with the health system and are attended by women regardless of delivery mode. Recruiting from these clinics facilitated inclusion of women with childbirth experiences across multiple facility types, including those who delivered at smaller local facilities, and reduced reliance on recruitment from labor wards alone.

**Fig. 1:**
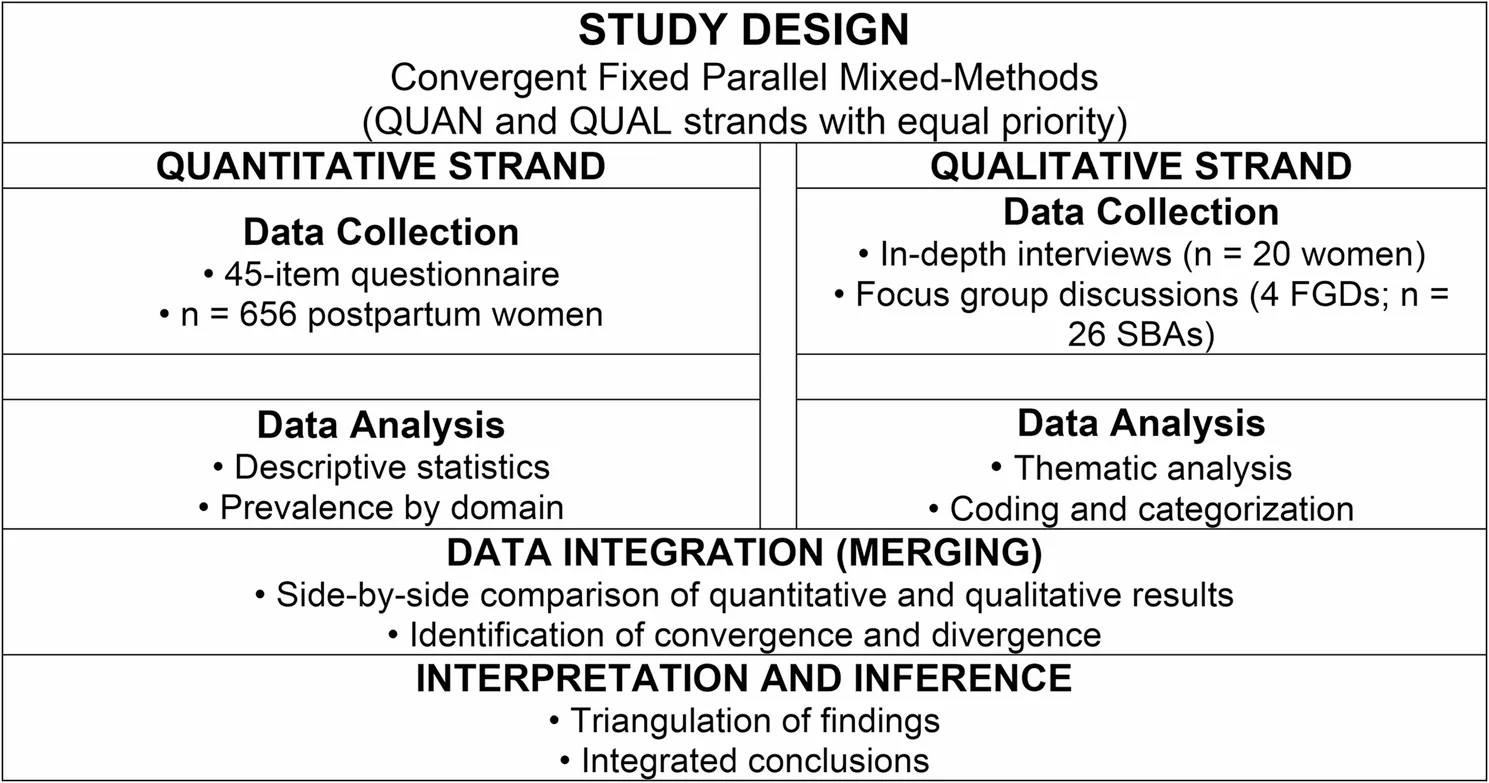
Quantitative and qualitative data were collected concurrently, analyzed independently with equal priority, and integrated during interpretation to examine mistreatment during childbirth from women’s and skilled birth attendants’ perspectives

**Fig. 2:**
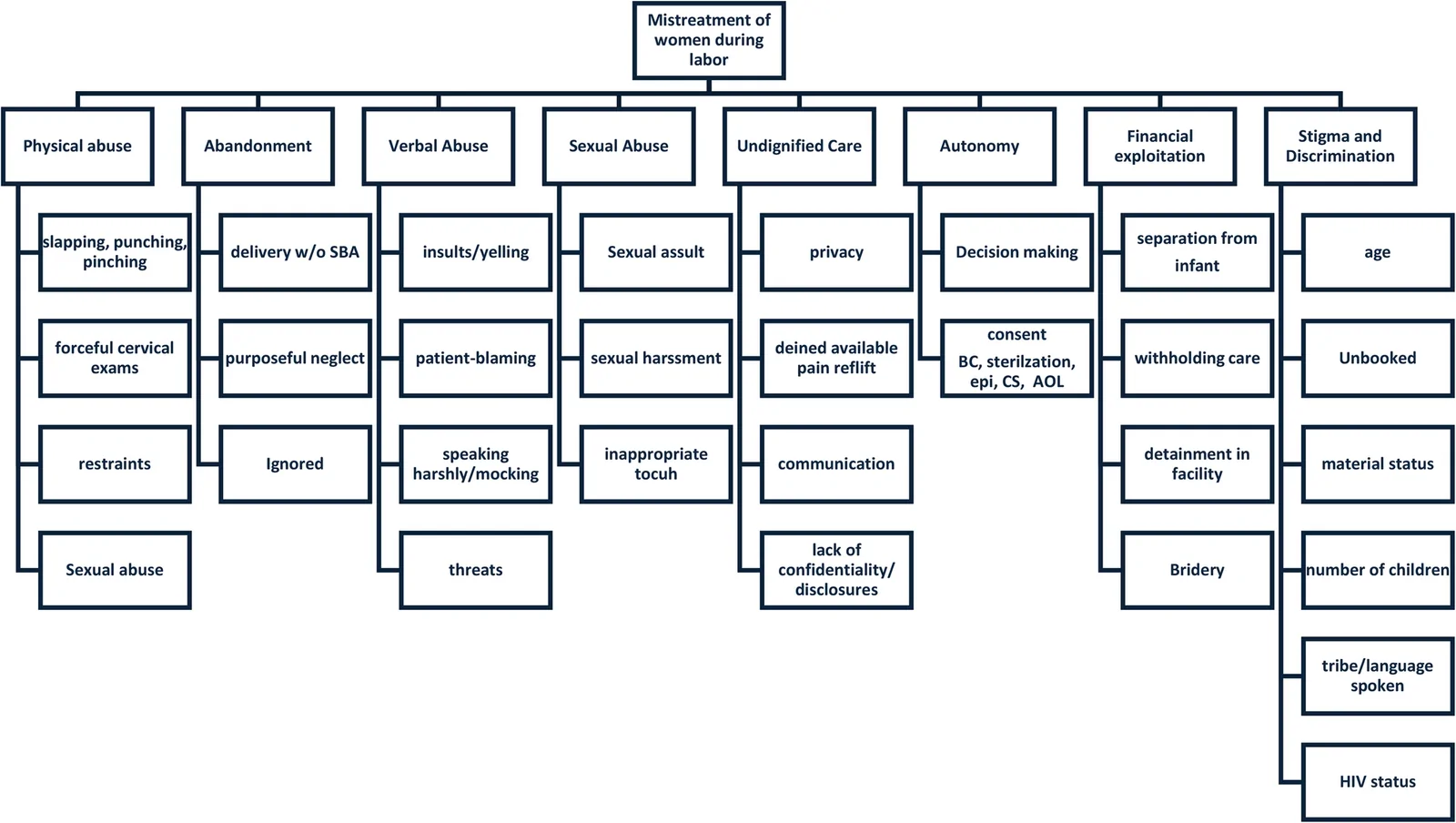
Conceptual framework developed through integration of Borhen et al. and Browers & Hill’s typology of mistreatment during childbirth with qualitative insights from Benin City, Nigeria, illustrating the dynamic interaction between individual, interpersonal, and systemic factors shaping women’s birthing experiences

This study was guided by a multidimensional conceptual framework that positions mistreatment during childbirth as a systems-level phenomenon operating across interpersonal, institutional, and structural domains (Fig. 1). The framework draws on the mistreatment typologies describe ed by Bohren et al. and Brower & Hill, informed by qualitative literature from Benin City to ensure contextual relevance [14, 17, 35]. This framework informed development of the survey instrument, qualitative interview guide, and mixed-methods integration strategy (Fig. 2).

### Quantitative component

#### Participants and eligibility criteria

Postpartum women were eligible to participate if they: (1) delivered in a healthcare facility with a skilled birth attendant in Benin within the past 12 months; (2) resided in Benin City; (3) were aged 18–65 years; (4) were able to communicate in English; and (5) were able to provide informed consent.

Women were excluded if they were unable to provide informed consent because of cognitive impairment or severe illness. Women who were medically unstable at the time of recruitment were not approached. The inclusion of women who delivered by cesarean section was intentional to capture the full spectrum of facility-based childbirth experiences. Questionnaire items that were not applicable to cesarean deliveries were coded as “not applicable” and excluded from item-level analyses.

#### Sampling and recruitment

Participants were recruited using consecutive purposive sampling from child immunization clinics at the three participating hospitals. Eligible women were identified and screened in person by trained research assistants using a standardized eligibility checklist. Prior to enrollment, each eligible participant was informed of the study’s purpose, procedures, potential risks including emotional discomfort, the voluntary nature of participation, and the right to withdraw at any time without consequence to her care. Verbal informed consent was obtained and documented by the research assistant on a standardized consent log. Verbal rather than written consent was selected to minimize participant burden and reduce barriers to participation in a survey-based study, consistent with the protocol approved by all participating institutional review boards.

Because recruitment occurred through postpartum immunization clinics rather than delivery wards, participants included women who had delivered at a variety of healthcare facilities throughout Benin City, including facilities other than the recruitment site.

No formal sample size calculation was conducted because the study was exploratory and aimed to estimate the prevalence and patterns of mistreatment during childbirth. Recruitment continued throughout the study period until approximately 650 completed surveys were obtained. This target sample size was established a priori and informed by sample sizes reported in previous studies of mistreatment during childbirth conducted in similar low- and middle-income country settings. The target was considered sufficient to estimate prevalence and explore associations between mistreatment and participant characteristics.

#### Data collection and measures

Data collection was supported by eight trained female research assistants who were independent of the participating facilities and not involved in clinical care. Research assistants received training in study procedures, ethical conduct of human subjects research, informed consent, confidentiality, and administration of the survey instrument.

A 45-item structured questionnaire was developed specifically for this study and is provided in Additional File 1. The instrument was informed by established mistreatment typologies and contextualized using prior qualitative work conducted in Benin City [14, 17, 35]. Mistreatment was categorized into eight domains: physical abuse, verbal abuse, sexual abuse, financial exploitation, lack of autonomy, undignified care, stigma and discrimination, and abandonment/neglect (Table 1).

**Table 1:** Operational definitions of the eight domains of mistreatment during childbirth as applied in this study

Variable	Operational Definition
Mistreatment (overall)	A woman was classified as having experienced mistreatment if she reported at least one event across any of the eight domains during childbirth.
Physical Abuse	The use of physical force against a woman, including slapping, hitting, pinching, or other forms of physical aggression during childbirth.
Verbal Abuse	The use of harsh, demeaning, threatening, or insulting language directed at a woman during childbirth.
Sexual Abuse	Any actual or threatened sexual act or inappropriate physical contact, occurring under conditions of coercion, force, or unequal power.
Financial Exploitation	The imposition of informal payments or financial barriers to care, including detention, delayed discharge, or withholding of services due to inability to pay.
Lack of autonomy	Failure to respect a woman’s right to make informed decisions about her care, including absence of informed consent, coercion, or exclusion from decision-making.
Undignified care	Treatment that undermines a woman’s dignity, including humiliation, disrespect, lack of privacy, or non-confidential care.
Stigma and Discrimination	Differential or inequitable treatment based on a woman’s personal, social, or health-related characteristics (e.g., age, ethnicity, parity, or medical history).
Abandonment/neglect	Failure to provide timely, continuous, or responsive care during childbirth, including ignoring requests for assistance or leaving a woman unattended.

Definitions were adapted from established frameworks on respectful maternity care and mistreatment during childbirth

Demographic and clinical variables collected included age, parity, marital status, educational attainment, household income, religion, ethnicity, antenatal care attendance, mode of delivery, and place of delivery.

Content validity was established through expert review by six maternal health specialists. Cognitive interviews were conducted with five postpartum women to assess clarity and comprehension, followed by pilot testing with an additional five postpartum women. Feedback from these activities informed iterative revisions to the questionnaire. Although formal psychometric validation was not conducted, the instrument was developed using established frameworks and underwent multiple stages of refinement.

Surveys were administered electronically using Google Forms on password-protected tablets in private areas of immunization clinics. Google Forms was selected because it allowed reliable data collection in settings with intermittent internet connectivity during fieldwork. Eligible participants were approached individually, and surveys were completed away from clinical staff and other patients whenever possible to promote privacy and minimize response bias. Responses were subsequently exported and stored in a secure REDCap database for data management and analysis. No identifying information was included in analytical datasets.

### Qualitative component

#### Participants and eligibility criteria

SBAs were eligible if they were actively practicing in Benin City, aged 18–65 years, able to communicate in English, and provided informed consent. Skilled birth attendants were defined as trained physicians, nurses, or midwives competent in managing normal pregnancy, childbirth, and the immediate postpartum period and in identifying and managing or referring obstetric complications.

#### Sampling and recruitment

Postpartum women were purposively selected for in-depth interviews (IDIs) to provide diverse perspectives on childbirth experiences. SBAs were recruited for focus group discussions (FGDs) using snowball sampling to ensure representation from multiple professional cadres, including physicians, nurses, and midwives. To minimize potential hierarchical influences on discussion, physicians participated in separate FGDs from nurses and midwives.

Sampling continued until thematic saturation was reached, defined as the point at which no new themes emerged from successive interviews and discussions. Final qualitative participants included 20 postpartum women and 26 SBAs (12 physicians and 14 nurses/midwives) who participated in four FGDs conducted separately by professional cadre.

#### Data collection

Semi-structured interview guides were developed to explore experiences of facility-based childbirth, perceptions and definitions of mistreatment, communication and informed consent, expectations of respectful maternity care, and health-system factors influencing care experiences.

All IDIs and FGDs were conducted by the principal investigator, an obstetrics and gynecology resident with formal training in qualitative research. Interviews and focus groups were conducted in private or semi-private locations within participating facilities, lasted approximately 45 min, and were audio-recorded with participant consent. Prior to each interview and focus group discussion, participants were informed of the study’s purpose, the topics to be discussed, the voluntary and confidential nature of participation, the audio-recording process, and their right to withdraw or decline to answer any question. Verbal informed consent was obtained and documented on a standardized consent log approved by the institutional review boards.

Interviews were conducted primarily in English, with occasional use of Nigerian Pidgin English to facilitate communication and participant comfort. Audio recordings were transcribed verbatim and de-identified prior to analysis.

### Reflexivity

The principal investigator is an obstetrics and gynecology resident with prior clinical and research experience in maternal health in Nigeria. Reflexive memo-writing, peer debriefing, and multidisciplinary team discussions were used throughout data collection and analysis to minimize the influence of pre-existing assumptions on interpretation.

### Data analysis

#### Quantitative analysis

Quantitative data were analyzed using *Stata SE 18* (StataCorp, College Station, TX). Descriptive statistics summarized participant characteristics and the prevalence of mistreatment overall and by domain.

Bivariate analyses were conducted to assess the association between reported mistreatment (overall and by domain) and selected sociodemographic and obstetric characteristics. Associations were tested using Pearson’s chi-square tests, and statistical significance was defined as *p* < 0.05. Multivariable logistic regression was not conducted due to the exploratory nature of the study and the very high prevalence of the outcome, which may violate model assumptions and limit interpretability due to reduced outcome variability.

#### Qualitative analysis

Qualitative analysis followed an iterative approach informed by constructivist grounded theory [36]. Transcripts were coded in NVivo 14 (QSR International) by a single investigator (D.E.) using a combination of open and focused coding. Codes were organized into a structured codebook and refined iteratively throughout the analytic process.

To enhance rigor, emerging themes and interpretations were reviewed through peer debriefing with senior multidisciplinary team members. The codebook and thematic framework were regularly discussed and refined to improve credibility and consistency.

#### Mixed-methods integration

Quantitative and qualitative data were analyzed independently and integrated during interpretation. A joint display matrix was used to compare quantitative prevalence estimates with qualitative themes across mistreatment domains. Findings were examined for convergence, divergence, and complementarity to provide a more comprehensive understanding of mistreatment during childbirth and its underlying drivers.

## Results

### Quantitative data results

#### Participant characteristics

A total of 656 postpartum women completed the quantitative survey. Women were aged 18–48 years (mean 30 ± 5.1). Of these, 296 (45.1%) were recruited from the University of Benin Teaching Hospital immunization clinic, 155 (23.6%) from Edo Specialist Hospital, and 205 (31.3%) from St. Philomena Catholic Hospital. Most participants were married (97%), Christian (98.2%), and of Bini (28.8%) or Igbo (17.4%) ethnicity. Nearly half delivered at public tertiary facilities (44.7%), while 21.2% delivered at private or secondary-level hospitals (Table 2).

**Table 2:** Distribution of participants’ sociodemographic and obstetric characteristics according to whether mistreatment during childbirth was reported, Benin City, Nigeria

Demographic	Total *N* (%)	Mistreatment not reported	Mistreatment reported	*p*-value
	656	*N* = 69	*N* = 587	
Age				0.450
18–25	114 (17%)	13 (18.8%)	101 (17.2%)	
26–35	431 (66%)	41 (59.4%)	390 (66.4%)	
36–50	111 (17%)	15 (21.7%)	96 (16.4%)	
Relationship status				0.510
Single/dating	20 (3%)	3 (4.3%)	17 (2.9%)	
Married	636 (97%)	66 (95.7%)	570 (97.1%)	
Highest education level				**0.046**
Primary school or less	18 (3%)	3 (4.3%)	15 (2.6%)	
Secondary school	253 (39%)	35 (50.7%)	218 (37.1%)	
University or more	385 (59%)	31 (44.9%)	354 (60.3%)	
Household Income (₦/month)				0.130
0-30k a month	117 (18%)	19 (27.5%)	98 (16.7%)	
30k-60k a month	200 (30%)	19 (27.5%)	181 (30.8%)	
60k to 150k a month	206 (31%)	21 (30.4%)	185 (31.5%)	
150k- 300k a month	92 (14%)	9 (13.0%)	83 14.1%)	
> 300k a month	41 (6%)	1 (1.4%)	40 (6.8%)	
Religion				0.230
Christianity	644 (98%)	69 (100.0%)	575 (98.0%)	
Islam/other	12 (20%)	0 (0.0%)	12 (2.0%)	
Tribe				0.420
Igbo	114 (17%)	11 (15.9%)	103 (17.5%)	
Esan	72 (11%)	8 (11.6%)	64 (10.9%)	
Benin	189 (29%)	22 (31.9%)	167 (28.4%)	
Edo	72 (11%)	3 (4.3%)	69 (11.8%)	
All other tribes	209 (32%)	25 (36.2%)	184 (31.3%)	
Number of children				0.130
1–2	480 (73%)	44 (63.8%)	436 (74.3%)	
3–4	161 (25%)	22 (31.9%)	139 (23.7%)	
≥ 5	17 (3%)	3 (4.3%)	12 (2.0%)	
Number of antenatal visits				**0.020**
0–3 visits	19 (3%)	2 (2.9%)	17 (2.9%)	
4–7 visits	91(24%)	2 (2.9%)	89 (15.2%)	
≥ 8	546 (83%)	65 (94.2%)	481 (81.9%)	
Mode of delivery				**0.030**
C-section	227 (35%)	32 (46.4%)	195 (33.2%)	
SVD/ other	429 (66%)	37 (53.6%)	392 (66.8%)	
Levels of patient care				**0.052**
Tertiary Hospital (UBTH)	293 (45%)	39 (56.5%)	254 (43.3%)	
Private Hospital (St. Philomena)	224 (34%)	15 (21.7%)	209 (35.6%)	
Secondary Hospital (Edo specialist)	139 (21%)	15 (21.7%)	124 (21.1%)	

Values are presented as n (%). *P*-values were calculated using the chi-square test. Statistical significance was set at *p* < 0.05, with statistically significant values shown in bold

*SVD* Spontaneous vaginal delivery

Mistreatment scores differed significantly by facility type (ANOVA *p* = 0.049). Participants delivering at the secondary hospital reported the highest mean mistreatment score (3.5 ± 2.7), compared with the private hospital (2.9 ± 2.4) and tertiary hospital (2.8 ± 2.6). Post-hoc Bonferroni-adjusted comparisons demonstrated significantly higher mistreatment scores at the secondary hospital compared with the tertiary hospital (mean difference 0.6, *p* = 0.049). Significant differences were also observed for physical abuse (*p* = 0.025) and autonomy-related mistreatment (*p* < 0.001), with higher scores reported among women delivering at the secondary hospital.

#### Prevalence and domains of mistreatment

Overall, 587 women (89.5%) reported experiencing at least one form of mistreatment during childbirth. However, only 2.6% perceived or labeled their experiences as mistreatment when asked directly.

The most frequently reported domains were undignified care (70%) and loss of autonomy (48.2%), followed by physical abuse (37.5%) and abandonment or neglect (16.3%). Less frequently reported were verbal abuse (14%), financial abuse (9%), stigma or discrimination (5.6%), and sexual abuse (2.6%) (Table 3).

**Table 3:** Reported prevalence and manifestations of mistreatment across eight domains during childbirth among study participants in Benin City, Nigeria

Domains of mistreatment	Reported, *N* (%)	Reported Manifestations, *N* (%)
Physical Abuse	246 (37.5)	Vaginal laceration repair w/o anesthesia 167 (25.26)
		Slapped, hit, punched or pinched: 21 (3.2)
		Forcibly restrained: 21 (3.2)
		No permission prior to cervical examinations 103 (15.7)
Verbal Abuse	92 (14.0)	**SBA yelled or spoke harshly** **48 (7.32)**
		SBA insulted patient 37 (5.64)
		SBA threatened patient 12 (1.83)
		SBA spoke disrespectfully 42 (6.4)
		SBA blamed patient for negative infant outcome 16 (2.44)
		SBA blamed patient for negative maternal outcome 18 (2.74)
Abandonment/ neglect	107 (16.3)	**Felt neglected** **68 (10.37)**
		SBAs were not responsive 66 (10.06)
Sexual abuse	17 (2.6)	**Felt sexually harassed** **7 (1.07)**
		Felt sexual assaulted 5 (0.76)
		Felt touched inappropriately 6 (0.91)
Lack of Autonomy	316 (48.2)	**Excluded from medical decision- making (16.92)**
		No consent prior to medical procedures 98 (14.94)
		No consent prior to contraception 131 (19.97)
Undignified care	456 (70)	**Denied a support person** **320 (48.78)**
		No privacy during delivery 61 (9.3)
		No privacy during cervical examinations *N* = 84 (12.8%)
		Medical history discussed without confidentiality 60 (9.15)
		Denied pain relief 48 (7.32)
		SBA did not communicate clearly 132 (20.43)
Financial exploitation	59 (9)	**Separated from infant until payment made** **25 (3.81)**
		Informal payment (bride) required 7 (1.07)
		Personal belonging withheld until payment 7 (1.07)
		Discharged delayed until payment received 23 (3.51)
		Belongings held until payment received 7 (1.07)
		Medical services held until payment was received 23 (3.51)
Stigma and discrimination	37 (5.6)	**Felt discrimination against during labor childbirth** **14 (2.13)**
		Due to medical history 8 (1.22)
		Due to not receiving antenatal care 3 (0.46)
		Due to parity 6 (0.91)
		Due relationship status 5 (0.76)
		Due tribe/ ethnicity 5 (0.76)
		Due age 11 (1.68)

Values are presented as n (%). Percentages are calculated using the total sample (*N* = 656)

Domains are not mutually exclusive; participants may report multiple forms of mistreatment

*SBA* Skilled Birth Attendant

#### Sociodemographic and obstetric characteristics and their association with mistreatment

Bivariate analyses (Table 2) identified significant associations between reported mistreatment and several obstetric and sociodemographic characteristics.

Women who delivered vaginally were more likely to report mistreatment compared with those who underwent cesarean delivery (χ², *p* = 0.030). Higher educational attainment was also associated with reported mistreatment (χ², *p* = 0.046), with women holding university-level education reporting higher rates than those with lower educational attainment.

Antenatal care attendance was significantly associated with mistreatment (*p* = 0.020), with women attending ≥ 8 visits less likely to report mistreatment. However, mistreatment remained highly prevalent across all levels of antenatal attendance.

No statistically significant associations were observed between mistreatment and age, parity, income, religion, or tribal affiliation (all χ² tests, *p* ≥ 0.05).

### Qualitative data results

Twenty postpartum women participated in IDIs, representing a range of childbirth experiences across public and private healthcare facilities in Benin City. Twenty-six skilled birth attendants participated in four FGDs, including 12 physicians and 14 nurses/midwives. Among the physicians, three were consultant obstetricians and nine were resident physicians. Focus groups were conducted separately by professional cadre to minimize potential hierarchical influences on discussion.

Qualitative analysis identified five major themes: (1) divergent expectations of care, (2) normalization of mistreatment, (3) communication and consent challenges, (4) health-system constraints, and (5) pathways to improving respectful maternity care (Table 4).

**Table 4:** Themes and subthemes reflecting the multifaceted nature of mistreatment during childbirth, highlighting women’s expectations, provider perspectives, and systemic contributors identified through qualitative interviews and focus group discussions

**Theme 1: Women’s Expectations**	
**Subthemes**	**Evidence**
Birth Companion	*“Some don’t actually understand what labor is*,* you see the young lady coming in into labor with her the whole family. By the time you say wait*,* She will say*,* “Oh*,*” they do not want my family to come in’ and our rooms although its a delivery suite*,* that provision that relatives should be there is not provided. The spouse can be there. By the time you tell them no*,* they feel is mistreatment. - FGD 3*,* participant1*,* female nurse/midwife*
Prenatal care	*“.if spoken to in some sort of harsh way or high pitch tone*,* she wouldn’t see that as some sort of maltreatment. She will know that*,* yes*,* this is necessary to deliver the baby. This sensitization will actually help prepare the patients and then put them in the best of behavior before they get to the doctors.” - FGD 4*,* participant 3*,* male physician*
Emotional Support	*“… the emotional support they need from their companions*,* their partners*,* they expect them from the doctors*,* you understand? “ For example*,* we had a patient in latent phase having contractions we had some student nurses available. She would tell them*,* rub my back*,* rub my back*,* please. I feel like this is where the husband should come in to help pet his wife” - FGD 4*,* Participant 2 male physician* *“They don’t have anything to do. What do they have to do? They don’t have anything. When a woman is in labor it’s for you to be petting her.” - IDI 12*,* postpartum woman*
Pain control	*“They don’t do much education*,* when it comes to pain medication*,* they don’t even offer you the choice of an epidural. I had my second child before I knew that they can actually give you an epidural in UBTH if you request it*,* I wasn’t aware of that*,* If they did much Education*,* women will be more aware*,* such to avoid*,* especially those ones that can be a pain*,* they will just opt for an epidural - IDI 16 postpartum woman*
Cost	*“if you don’t have money*,* you will not receive treatment*,* you buy your own drugs. So most patients*,* they go to general hospital thinking the government*,* will provide drugs for them. Getting here we will ask them to go and buy the drugs*,* but when they don’t have money to buy it *,* no treatment will be given. And they will think you are maltreating them” - FGD 1*,* participant 1*,* female nurse/midwife* *“.they would tell you do something*,* I will pay later. Here in the labor ward you cannot make blood available… you ask them to go make plans on how you get blood. They feel you are maltreating them because they’ve been giving the impression that when they get to University of Benin Teaching Hospital*,* everything is available to solve their immediate need.” - FGD 1*,* participant 1*,* female nurse/midwife*
**Theme 2: Uncooperative Patient**	
**Subthemes**	**Evidence**
Encouragement	*“there’s this tendency because you want to have a good outcome and you don’t want birth asphyxia you say ‘madam push’*,* and you can see some personnel in some centers*,* practically tapping the woman to encourage her ‘ you’re wasting time push’. So I think to some level in some cultures*,* that is acceptable and not considered physical abuse.” - FGD 2*,* participant 6*,* female physician*
Patients Best Interest	*“mistreatment can also physical abuse like hitting the patient*,* healthcare provider might not intend it as mistreatment. For example a woman that is not making adequate effort to bare and the midwife or doctor is concerned that the baby might be asphyxiated because of the positioning and is trying to hit her to madam push*,* don’t kill your baby or something like that. But physical beating in the in the process of making the woman to make effort can also be counted as mistreatment. - FGD 1*,* participant 5*,* female nurse/midwife* *“ there is a tendency as humans*,* not because we want to abuse the patient*,* but because you want the very best for the patient*,* when the patient is not cooperating*,* there’s that tendency*,* to want to shout at her*,* you want to maybe hit her in order for you to do the right thing. So the patient can deliver. So*,* these can put them at risk of being mistreated.” - FGD 2*,* participant 6*,* Female physician*
Behavior of Relatives	*“You will see patient relatives hitting the doctors that has happened. Most of the time doctors have to keep their cool and just take it. We have seen instances where the patient relative will even slap the doctor ‘why are you not doing something’ " - FGD 4*,* participant 3*,* male physician*
Self-Control	*“Maybe the woman will be shouting unnecessarily. There are some pains you’ll have to bear because you’re in labor. it’s not easy. But maybe the woman will be shouting unnecessarily. Maybe he (the doctor) is thinking about something*,* and the women distracts him. Maybe because of that*,* it will make the doctor hit the women.” -IDI 23 postpartum woman* *“If they are not corporative*,* like for instance*,* it’s not yet time for you to push. They are pushing because maybe they don’t know how to control it. It might irritate the doctors because they are giving them extra work. Because if you push when you are not supposed to push*,* you will get perineal tears. And that will result in a longer time for you staying in the theater. You have to suture*,* sometimes suturing is annoying depending on how the tear is.” -IDI 17postpartum woman*
**Theme 3: Mistreatment Define**	
**Subthemes**	**Evidence**
Women	*“Maybe they will tell you to do something and they will shout at you or they will ask you a lot of questions and quarrel with you.” - IDI 5 postpartum woman* *“Okay*,* I think shouting at them would be unacceptable. I know people express their pain differently*,* and sometimes it can be irritable to you. But as a professional*,* you should just compose yourself and show compassion regardless.” - IDI 16 postpartum woman* *“to have their questions unanswered*,* once they have questions they get ignored*,* or to ignore their pain*,* even when they are going through something and*,* you talk to them rudely like: “You are not the first to go through this”. Some persons do that.” - IDI 57 postpartum woman* *“Maybe if they did not attend to the woman on time ” - IDI 97 postpartum woman*
SBA	*“mistreatment can also be in terms of verbal response or verbal statement we make to the woman even while they are in labor*,* in terms of giving instructions*,* we might inadvertently shout at the patient and this could also go to affect them psychologically. ” - FGD 2*,* participant 6*,* female physician* *“you must seek consent before you do anything to me. If you disrespect me*,* that’s maltreatment” – FDG 3*,* participant 3*,* female nurse/midwife* *“when she’s feeling pain and you know that you alleviating the pain at that point in time will be what she needs or will not cause any harm. You denying her of that*,* would could also be termed as abuse.” - FDG 3*,* participant 1*,* female nurse/midwife* *“ when a patient is neglected*,* but we don’t do that here.if she’s calling for help*,* for attention*,* and you*,* walk away*,* you ignore the patient. That’s mistreatment” - FDG 3*,* participant 3*,* female nurse/midwife* *“Every patient in labor deserves attention*,* deserves explanation. You need to talk to your patient*,* to get her attention*,* explain each stage in order*,* for you to be able to get*,* you know. ehh*,* her compromising to whatever procedure you are carrying out. If you don’t do this*,* she’s asking you question and you are ignoring. It’s maltreatment. ” - FDG 3*,* participant 2*,* female nurse/midwife* *“an individual or a group of persons that lack the basic required knowledge and skill to manage labor and ease delivery or administration of inappropriate interventions and inappropriate timings in labor and delivery” - FDG 2*,* participant 7*,* male physician* *“like to narrow it down to down to basic empathy and compassion. I think when you deny you that*,* you are sort of mistreating them sort of.” - FDG 2*,* participant 7*,* male physician*
**Theme 4: SBA Burnout**	
**Subthemes**	**Evidence**
Payment/incentives	*“.the government should also see to improving the welfare of doctors because they cannot be over working a doctor and under paying doctors. Most of the time doctors in Nigeria are running away*,* the ones who still have the passion to want to practice*,* they are just biding their time*,* writing these foreign exams*,* just running away .” - FDG 4*,* participant 1*,* male doctor* *“They said a hungry man is an angry man. So if you are hungry and a patient tells you*,* nurse come and rub my waist and the other one is shouting nurse come and rub my waist*,* with empty stomach*,* you will not have that joy*,* I’m sorry to say.” - FGD 1*,* participant 5*,* Female nurse/midwife*
Understaffed	*“If we have more hands and there’s more manpower*,* I think it would significantly affect*,* overall treatment of patients. Because you can have one doctor treating 50 to 100 patients*,* and you expect him to have the same efficiency as one doctor handling 10 patients*,* obviously is going to be a different” - FDG 4*,* participant 2*,* male physician*
Undersupplies	*“… they should also renovate and equip the health sector in Nigeria. If we have more equipment*,* we have more health personnel and we are being given incentive*,* good salary*,* I think when someone is working*,* even if you are with one patient or two patients*,* you’ll be able to give quality care” - FDG 4*,* participant 2*,* male physician* *“…if doctors want to do something*,* they need enough tools. They need to be assured that those things can be made available to improve his treatment for that patient.” - - FDG 3*,* participant 1*,* female nurse/midwife*
SBA Attitude	*“patients prefer going to TBA for antenatal care*,* when they come here on emergency*,* they don’t abide by hospital rules and regulation*,* they want to do what they were doing at the TBA. Then they can get the healthcare provider angry*,* and then healthcare providers can talk to them in a way that they feel is being maltreated.” - FDG 1*,* participant 1*,* female nurse/midwife*
**Theme 5: Mistreatment Experienced**	
**Subthemes**	***Evidence***
Physical Abuse	*Respondent: it’s not really their fault* *Interviewer: you can tell me.* *Respondent: like all those hitting*,* open your leg*,* that kind of thing. -IDI 80 postpartum woman*
Verbal Abuse	*“…the nurses were now screaming like madam you have to push this child now*,*” IDI 17*,* postpartum woman* *“the nurse is expected to yell*,* because the baby was already coming out*,* so the nurses are expected to yell or hit her legs or help her out just to bring out the baby.” - IDI 99 postpartum woman*
Sexual Abuse	*“For a woman*,* woman to woman*,* when she was inserting her fingers to check my cervix*,* I wasn’t even prepared*,* and I felt that pain and it is still etched in my memory you understand for a woman*,* but when a doctor came*,* later a male doctor came afterwards he was so gentle.” - IDI 28 postpartum woman*
Abandonment	*“I needed somebody to check my progress*,* I always had to be calling because they left me alone*,* nobody was there with me so and I also needed water but nobody paid attention to me until the nicer ones came along.” - IDI 16 postpartum woman*
Lack of Autonomy	*“…from the labor room to the ward you’re not allowed to carry the baby the very first day of the CS you can only see your baby from where she’s crying or from where they’re taking care of the baby *,* you cannot hold her so for me it was it was traumatic.” - IDI 28 postpartum woman*
Undignified Care	*“What I would have wanted to change is for my husband to be with me at least*,* or anybody so I wouldn’t be alone.” - IDI 67 postpartum woman*
Financial Abuse	*“… the person*,* maybe they want to discharge you. They would ask you to go and pay your money. Maybe the money they ask you to pay is too big or you look for the money. They would ask you a lot of questions and say go and pay your money and go home. That’s not good now.” - IDI 5 postpartum woman*
Discrimination	*“sorry to say even when the patient is not cooperative*,* a cleaner in labor is not cooperative and doctor’s wife in labor is not cooperative. The attitude to this same uncooperative patients of different social class differs. The way you will shout on the cleaner*,* we know you will not be able to shout on the CMD’s wife that is not cooperative in labor also. So it still boils down to the social class and financial status or social capital of that person at the end of the day.” - FGD 2*,* participant 1*,* male physician*

Verbatim quotations were minimally edited for clarity without altering meaning

*IDI* In-depth Interview, *SBA* Skilled Birth Attendant

#### Theme 1: Divergent expectations of hospital-based childbirth between women and SBAs

This theme encompassed five subthemes: birth companions, emotional support, prenatal education, pain control, and cost of care.

Many women expressed a strong desire to have a birth companion present during labor and delivery. The inability to have such support was frequently described as a major source of dissatisfaction:

“What I would have wanted to change is for my husband to be with me at least, or anybody, so I wouldn’t be alone.” *– IDI 67*,* postpartum woman*.

SBAs acknowledged the importance of companionship but noted that systemic and spatial constraints made this difficult to implement:

“We lack space here… If we have enough space, we could have cubicles where women stay with their partners. But because we don’t have space, we don’t allow them to come in.” - *FGD 3*,* participant1*,* female nurse/midwife*.

In the absence of companions, women often expected SBAs to provide emotional support, though many SBAs felt unable to do so given workload constraints:

“They [SBAs] don’t have anything to do? When a woman is in labor, it’s for you [SBAs] to be petting (comforting) her.” -*IDI 12*,* postpartum woman*.

Both women and SBAs also highlighted the role of inadequate prenatal education in shaping unrealistic expectations and perceptions of mistreatment.

“If spoken to in a high tone, she wouldn’t see that as maltreatment if she was sensitized. This preparation helps put them in the best behavior before they meet the doctors.” – *FGD 4*,* participant 3*,* male* physician.

Women, in turn, voiced dissatisfaction with the limited information provided during antenatal care, especially regarding pain management options:

“They don’t do much education on pain medication. I didn’t even know you could get an epidural in [healthcare facility] until my second child.” – *IDI 16*,* postpartum woman*.

Lastly, both groups identified financial expectations as a frequent source of conflict and perceived mistreatment:

“If you don’t have money, you won’t receive treatment. We ask them to go and buy the drugs, but if they can’t, no treatment will be given, and they think you are maltreating them.” – *FGD 1*,* participant 1*,* female nurse/midwife*.

#### Theme 2: Some behaviors exhibited by women during childbirth are viewed as “uncooperative” by SBAs

Three subthemes were identified: self-control/coping, acting in the woman’s best interest, and encouragement.

Both women and SBAs described how perceptions that a women is not calm during labor sometimes triggered negative reactions from SBAs:

“There are some pains you’ll have to bear because you’re in labor. But if a woman is shouting unnecessarily, maybe the doctor gets distracted and hits her.” – *IDI 23*,* postpartum woman*.

Some SBAs rationalized shouting or physical contact as being in the woman’s best interest and to ensure positive neonatal outcomes:

“You don’t want to abuse the patient, but when she’s not cooperating, you might shout or hit her so she can deliver safely.” – *FGD 2*,* participant 6*,* female* physician.

Others described such behavior as a culturally accepted form of encouragement:

“You might tap the woman and say, ‘You’re wasting time, push!’ In some cultures, that’s acceptable and not considered abuse.” – *FGD 4*,* participant 8*,* male physician*.

#### Theme 3: Mistreatment during childbirth defined

Two subthemes emerged: definitions by women and definitions by SBAs.

Women frequently defined mistreatment as verbal abuse, neglect, or poor communication:

“Shouting at them would be unacceptable… as a professional, you should just compose yourself and show compassion.” – *IDI 16*,* postpartum woman*.

Similarly, SBAs described mistreatment as encompassing verbal abuse, neglect, lack of informed consent, substandard care, and failure to manage pain:

“When you deny empathy and compassion, you are mistreating them.” - *FGD 2*,* participant 3*,* male physician*.

#### Theme 4: Burnout among SBAs and effects on patient care

Four subthemes were identified: payment, understaffing, undersupply, and attitude.

SBAs consistently expressed frustration about being underpaid and overworked, noting how these conditions influenced their behavior toward patients:

“They say a hungry man is an angry man. If you’re hungry and two patients are shouting for you to rub their waists, with an empty stomach, you won’t have that joy.” – *FGD 1*,* participant 5*,* female nurse/midwife*.

They also described how inadequate infrastructure and staffing reduced morale and quality of care:

“If we had more equipment, more health personnel, and good salaries, even with two patients, you’d be able to give quality care.”- *FGD 1*,* participant 5*,* female nurse/midwife*.

#### Theme 5: Women and SBAs accounts of mistreatment women experience during childbirth

Eight subthemes were identified: physical abuse, verbal abuse, sexual abuse, financial abuse, abandonment, lack of autonomy, undignified care, and discrimination.

Several women recounted incidents of physical and verbal abuse:

“They hit me to open my legs.” – *IDI 80*,* postpartum woman*.

“The nurses were screaming at me, ‘Madam, you have to push this child now!’” – *IDI 17*, *postpartum woman*.

Some described sexual abuse and violations of autonomy:

“When she was inserting her fingers to check my cervix, I wasn’t even prepared, and I still remember that pain.” – *IDI 28*,* postpartum woman*.

Others reported being abandoned or denied dignified care:

“I always had to call because they left me alone. Nobody was there until the nicer ones came along.” – *IDI 16*,* postpartum woman*.

Women also described financial abuse when unable to pay hospital fees:

“They’d say, ‘Go and pay your money and go home.’ That’s not good.” – *IDI 5*,* postpartum woman*.

Finally, both women and SBAs highlighted discrimination based on social class:

“Even when patients are not cooperative, the way you shout at a cleaner in labor is not how you’d shout at the doctor’s wife. It boils down to social class.” – *FGD 2*,* participant 1*,* male physician*.

Qualitative findings supported quantitative results, particularly the high prevalence of undignified care and loss of autonomy, while also explaining the discrepancy between high reported mistreatment and low self-identification through normalization and systemic constraints.

### Mixed- method integration

Overall, findings demonstrated strong convergence. Quantitatively, undignified care (70.0%) and loss of autonomy (48.2%) were the most frequently reported forms of mistreatment. These findings aligned with qualitative accounts in which women described non-consented procedures, limited communication, and exclusion from decision-making. SBAs attributed these experiences to structural constraints, including high workload, staffing shortages, and inadequate infrastructure.

Similarly, reported physical abuse (37.5%) and neglect or abandonment (16.3%) corresponded with qualitative descriptions of coercive labor practices, delayed provider response, and intermittent bedside care. While women described these experiences as mistreatment, SBAs often contextualized them as responses to perceived obstetric urgency and resource limitations.

Divergence was observed in labeling: although 89.5% of women reported at least one form of mistreatment, only 2.6% identified their experience as such. Qualitative findings explained this gap through normalization of poor-quality care, limited awareness of respectful maternity care standards, and hierarchical patient–provider relationships.

Complementarity was also evident. While quantitative data quantified the burden of mistreatment, qualitative findings explained its underlying mechanisms, including burnout, system constraints, and power dynamics within maternity care settings.

Together, the integrated findings indicate that mistreatment is a structurally embedded and normalized feature of facility-based childbirth rather than isolated provider behavior.

## Discussion

This mixed-methods study provides comprehensive evidence that mistreatment during facility-based childbirth in Benin City, Nigeria, is highly prevalent and shaped by intersecting interpersonal and structural factors. By integrating large-scale prevalence data with in-depth accounts from both women and skilled birth attendants (SBAs), the findings demonstrate that mistreatment reflects routine practices embedded within health system constraints rather than isolated acts of individual misconduct.

Nearly nine in ten women reported experiencing at least one form of mistreatment, yet only a small minority explicitly labeled their experience as such. Integration of qualitative findings helps explain this divergence. While women and SBAs articulated similar definitions of mistreatment including verbal abuse, neglect, lack of informed consent, inadequate pain management, and physical coercion these behaviors were often described as normalized within the realities of busy labor wards. This suggests that mistreatment may be routinized within facility-based childbirth, occurring in ways that are recognizable but not consistently framed as violations of respectful care standards. Limited rights-based awareness, constrained patient agency within hierarchical systems, and prioritization of neonatal outcomes over maternal experience may contribute to this normalization. These findings are consistent with prior studies in Nigeria [18, 21, 25, 37, 38].

Quantitative findings identified undignified care and loss of autonomy as the most frequently reported domains. Qualitative data further revealed how these experiences were shaped by systemic factors, including space limitations restricting birth companions, inadequate prenatal counselling regarding pain management, financial barriers to care, and workforce shortages. SBAs described chronic understaffing, insufficient supplies, and burnout as key drivers of strained patient–provider interactions. These findings align with prior research in sub-Saharan Africa identifying structural resource constraints and provider fatigue as contributors to disrespectful care [26–28].

Importantly, mistreatment emerged not solely from negative provider intent but from misaligned expectations and institutional pressures. Some SBAs described shouting or physical prompting as necessary to ensure neonatal safety, reflecting deeply ingrained norms that prioritize clinical outcomes over patient experience. Women, meanwhile, expressed expectations for emotional support and communication that were often unmet within high-volume facilities. These bidirectional frustrations underscore that respectful maternity care cannot be achieved through individual-level behavior change alone.

The alignment between high prevalence of undignified care and autonomy violations and qualitative accounts of restricted companionship, limited consent processes, and hierarchical communication suggests that these experiences are structurally embedded within routine care delivery rather than episodic deviations from standard practice.

Policy implications extend beyond provider training. These findings indicate that respectful maternity care cannot be achieved through provider-focused interventions alone. Structural reforms including workforce expansion, infrastructure redesign to support birth companionship, integration of respectful maternity care indicators into routine quality monitoring, strengthened accountability mechanisms, and incorporation of rights-based counselling into antenatal care are essential. Without addressing these systemic constraints, improvements in clinical care may not translate into improved patient experience.

### Strength and limitations

Strengths include the large quantitative sample, inclusion of both women’s and providers’ perspectives, and use of a convergent mixed-methods design allowing systematic integration of data strands. Inclusion of SBAs provides insight into supply-side constraints often absent from prevalence studies.

Limitations include the absence of screening and refusal logs during participant recruitment. Consequently, the number of women assessed for eligibility, declining participation, and site-specific participation rates could not be determined, limiting assessment of potential selection bias. The study also relied on self-reported experiences, which may be influenced by recall or social desirability bias. The survey instrument, while developed from established typologies and refined through expert review, cognitive interviews, and pilot testing, was not formally psychometrically validated in this setting. Participation was restricted to women able to communicate in English, which may limit generalizability to non-English-speaking populations. The study was conducted in urban facilities in Benin City, potentially limiting generalizability to rural contexts. Additionally, the cross-sectional design precludes causal inference regarding determinants of mistreatment, and qualitative analysis was conducted by a single coder, which may have introduced interpretive bias despite the use of reflexive memo-writing, peer debriefing, and multidisciplinary review.

## Conclusion

Mistreatment during childbirth in Nigeria is highly prevalent and shaped by systemic health system constraints. Integration of women’s and providers’ perspectives demonstrates that disrespectful care is sustained by workforce shortages, institutional norms, and misaligned expectations rather than isolated acts of misconduct. The discrepancy between reported experiences and recognition of mistreatment suggests normalization of poor-quality care within facility-based childbirth. Efforts to improve maternal health outcomes must therefore address both system capacity and accountability, ensuring that clinical safety and respectful care are jointly prioritized. Such approaches are essential to ensure that increased facility-based delivery translates into both improved clinical outcomes and positive childbirth experiences.

## Supplementary Information

Supplementary Material 1

## Data Availability

The datasets generated and analyzed during the current study are not publicly available due to the sensitive nature of participants’ experiences but are available from the corresponding author on reasonable request. De-identified quantitative data and qualitative codebooks can be shared with researchers who meet the criteria for access to confidential data.
