# Supplementary material for: Mistreatment during facility-based childbirth in Benin City, Nigeria: A convergent mixed-methods study of prevalence and drivers
Source: BMC Pregnancy Childbirth. 2026 Aug 10;26:1275. doi: 10.1186/s12884-026-09541-z (PMC13737790; doi:10.1186/s12884-026-09541-z)
Supplement: Supplementary file 1 [file 12884_2026_9541_MOESM1_ESM.docx]

# Additional File 1

## Postpartum Mistreatment During Childbirth Questionnaire

Instructions to Participants: Please answer the following questions based on your most recent childbirth experience at this facility. For Sections 2–8, responses are Yes or No unless otherwise indicated.

### Section 1: Demographic and Obstetric Characteristics

1. Age (years): 18–24; 25–34; 35–40; 41 or older

2. Marital status: Single; Married; Partnered; Widowed

3. Religion: Christianity; Islam; Traditional religion; Other (specify)

4. Occupation: Employed; Self-employed; Unemployed; Student; Other (specify)

5. Highest level of education completed: No formal education; Primary school; Secondary school; University or higher

6. Antenatal care attendance for this pregnancy: Attended one or more antenatal visits; Did not attend any antenatal visits

7. Mode of delivery: Spontaneous vaginal delivery; Operative vaginal delivery; Cesarean section

8. Location of previous delivery (if applicable): Health care facility; Traditional birth attendant; Church; Not applicable

9. Previous mode of delivery (if applicable): Spontaneous vaginal delivery; Operative vaginal delivery; Cesarean section; Not applicable

10. Length of stay after delivery: Less than 24 hours; 1–2 days; 3 days or more

### Section 2: Experiences During Labor and Delivery (Yes/No Responses)

#### A. Physical Abuse

11. Were you slapped, hit, punched, or pinched during labor or delivery?

12. Were you physically restrained against your will?

13. Did you undergo a vaginal examination without your permission?

14. Was a vaginal tear repaired without numbing medication?

#### B. Abandonment or Neglect

15. When you called for a healthcare provider, were they responsive?

16. Did you feel neglected or ignored during labor or delivery?

17. Was a skilled birth attendant present at the time of your birth?

#### C. Verbal Abuse

18. Were you insulted or verbally abused during labor?

19. Did anyone yell at you or speak harshly to you?

20. Were you threatened with withholding care?

21. If you or your baby experienced a complication, were you blamed for the outcome?

22. Were you spoken to respectfully by healthcare providers?

#### D. Sexual Abuse

23. Were you touched in a way that made you feel uncomfortable or inappropriate?

24. Were you sexually harassed during labor or delivery?

25. Were you sexually assaulted during labor or delivery?

#### E. Undignified Care

26. Did healthcare providers clearly explain your condition and care?

27. Were you mocked, shamed, or belittled during labor or delivery?

28. If you requested pain relief, were you denied pain relief? (Skip if you did not request pain relief)

29. Was your medical information discussed where others could hear without your consent?

30. Did you have privacy during vaginal examinations and delivery?

31. Were you allowed a support person during labor and delivery?

#### F. Autonomy and Informed Consent

32. Was your permission obtained before medical procedures (e.g., cesarean section, episiotomy, induction)?

33. Were you given contraception after delivery? If yes, did you consent to receiving contraception?

34. Were you involved in decisions about your labor and medical care?

#### G. Financial Abuse

35. Were services withheld until payment was made?

36. Was your discharge delayed due to inability to pay?

37. Were your personal belongings held until payment was made?

38. Were you separated from your baby due to unpaid bills?

39. Were you asked to pay a bribe to receive care?

#### H. Stigma and Discrimination

40. Were you treated poorly because of your tribe or native language?

41. Were you treated poorly because of your income?

42. Were you treated poorly because of your marital status?

43. Were you treated poorly because of the number of children you have?

44. Did you experience discrimination at any time during labor or delivery?

45. If you did not attend antenatal care, were you treated differently upon admission?
